# How has Radical Right Support Transformed Established Political Conflicts? The Case of Austria

**DOI:** 10.1080/01402382.2013.814956

**Published:** 2013-08-05

**Authors:** Julian Aichholzer, Sylvia Kritzinger, Markus Wagner, Eva Zeglovits

## Abstract

*In many European party systems, the radical right has challenged established patterns of political competition. This article studies the consequences of this by using the case of the Freedom Party of Austria (FPÖ) and data from Austria’s first national election study (AUTNES). It is found that the FPÖ has weakened Austria’s previously highly stable system of socio-structural and ideological divisions as expressed by the two mainstream parties, the People’s Party and the Social Democrats. In socio-structural terms, the FPÖ has undermined the Social Democrats’ support base. In ideological terms, FPÖ voters have distinct views on newer issues such as immigration, European integration and dissatisfaction with the political system, but its supporters’ views on Austria’s traditional conflicts surrounding the economy and social and religious values cannot explain the party’s success. These findings further our understanding of the transformation of political conflicts not just in Austria, but in Western Europe in general*.

Over the past two decades, radical-right parties have become a well-established feature of many European party systems.^1^ Their rise has been accompanied by extensive scholarly work that has sought to define, document and explain the phenomenon (e.g. Betz 1994; Bornschier 2010, 2012; Kitschelt 1995, 2007; Luther 2011; McGann and Kitschelt 2005; Mudde 2007; Norris 2005; Rydgren 2005).

Two perspectives dominate the effort to understand support for the radical right and its repercussions for other political parties and for party competition more generally. First, scholars have tried to understand how the success of the radical right reshapes party competition and thus threatens established political parties. Here, particular emphasis has been placed on the socio-structural make-up of support for the radical right, with research generally finding that, in broad terms, radical-right parties gain votes among working-class and lower middle-class voters, social groups that used to vote for centre-left and left parties (e.g. Ivarsflaten 2005; Kriesi *et al*. 2008; Lubbers *et al*. 2002; Oesch 2008; Rydgren 2007).

Second, scholars have investigated the ideological and attitudinal drivers of radical-right support. Here it has been shown that the political views that underlie radical-right support are related to newly emerging values and conflicts over issues such as immigration and European integration (e.g. Bornschier 2010, 2012; Cutts *et al*. 2011; van der Brug and Fennema 2007). In contrast, support for the radical right generally depends relatively little on traditional ideological conflicts over the economy or religion (e.g. Mudde 2007; Rovny 2013; Wagner and Kritzinger 2012).

In this paper we use both of these approaches to understand the consequences of the rise of the radical right for political competition. By integrating these two theoretical perspectives of research on the radical right we aim to provide an encompassing picture of how support for the radical right has transformed established political conflicts. We therefore link the extent to which the radical right challenges pre-existing socio-structural and ideological divisions with how it brings to the fore important new political conflicts.

We do so by analysing Austria, which is a convenient case study due to the simplicity both of its party system and of its traditional socio-structural and ideological divisions (Müller 1997). Moreover, it has one of the most prominent radical-right parties in Europe, the Austrian Freedom Party (*Freiheitliche Partei Österreichs*, FPÖ). This party has had continued electoral success since 1986, averaging around 15 per cent of the vote in national parliamentary elections and never falling below the 9.7 per cent of 1986. It is also one of a small number of radical-right parties to have participated in government (de Lange 2012; Zaslove 2012).^2^ Yet we know relatively little about how the rise of the FPÖ has reshaped Austrian politics, which previously had been one of the most stable and well-structured European party systems (Müller 2006). In this paper, our aim is to contribute to our understanding of how political competition has been transformed in Austria by examining why some Austrians choose to support the radical right FPÖ and how FPÖ supporters differ from those of the two Austrian mainstream parties, the Austrian People’s Party (*Österreichische Volkspartei*, ÖVP), part of the Christian Democratic party family, and the Austrian Social Democrats (*Sozialdemokratische Partei Österreichs*, SPÖ). Furthermore, as this case illustrates how a radical-right party relates to pre-existing lines of conflict while opening up new axes of polarisation, the results are important for understanding changes in the structure of political competition outside of Austria as well. Such changes in the issue structure of party systems can have important consequences for how parties compete as they may reshuffle the electorate and restructure the saliency of issue positions (e.g. Carmines and Stimson 1986; de Vries and Hobolt 2012; Riker 1986; Schattschneider 1960).

In this paper we show that the radical right in Austria challenges the country’s traditional – yet still important – structure of party competition by undermining the socio-structural base of support for the Social Democrats and by mobilising supporters through new political conflicts that challenge both mainstream parties. The traditional socio-structural divisions were class, religion and urban versus rural residence, and on these attributes Austrian radical-right supporters largely resemble Social Democratic supporters and are only different from People’s Party supporters. The traditional ideological conflicts linked to these socio-structural divisions were based on the economy on the one hand and social and religious values on the other (Müller 1997; Plasser *et al*. 1992). Here, the positions of FPÖ supporters are weakly defined and do not help us to explain the FPÖ’s success. Instead, in ideological terms support for the radical right is primarily explained by where citizens stand on new political conflicts, specifically immigration, European integration and anti-elite sentiments. We find that on these issues, FPÖ supporters differ from the supporters of both mainstream parties, i.e. the SPÖ and ÖVP.^3^

Our study takes advantage of the new Austrian National Election Study (AUTNES 2009), which for the first time allows a full analysis of the drivers of support of the FPÖ in relation to the two mainstream parties SPÖ and ÖVP. The survey contains detailed questions on socio-structural attributes as well as on attitudes related to established and new political conflicts. Previous single-country case studies of FPÖ voting relied on results from non-academic surveys (e.g. McGann and Kitschelt 2005) or exit polls (e.g. Plasser *et al*. 2007), yet such surveys are brief, containing a limited set of attitudinal questions. While comparative studies on radical-right support that included Austria as one of their cases have used the 2002/3 European Social Survey (ESS) (e.g. Arzheimer and Carter 2009; Ivarsflaten 2008; Lucassen and Lubbers 2012; Norris 2005; Oesch 2008; Rydgren 2008), they are hampered by the fact that the early 2000s were a period when the FPÖ was unusually unpopular due to its participation in government as a junior partner (Heinisch 2003). This means that there are relatively few FPÖ supporters in the EES sample and that the supporters that are included may be different from the FPÖ’s supporters more generally over the past 25 years.

The paper is structured as follows. First, we briefly describe the Austrian setting before considering how support for radical-right parties is linked to established socio-structural divisions and ideological conflicts as well as to new political issues. We then describe our data and our method of analysis before presenting the results. Our conclusion sums up our findings and considers their implications.

## The FPÖ’s Electoral Success

**Figure 1:**
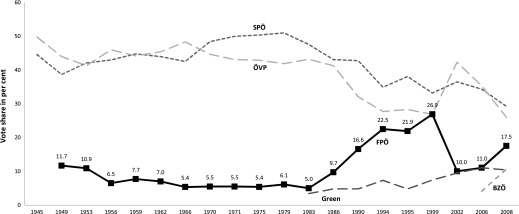
ELECTION RESULTS IN AUSTRIA SINCE 1945 *Note*: FPÖ in 1949 and 1953: results of its predecessor, the *Verband der Unabhängigen*; Greens in 1983: results of its two predecessors, the *Vereinigte Grüne Österreichs* and the *Alternative Liste Österreichs*; data from the Austrian Federal Ministry of the Interior, available at http://www.bmi.gv.at/cms/bmi_wahlen (accessed 22 August 2012).

Austria’s FPÖ is one of the most successful radical-right parties in Europe. In the three decades before Jörg Haider took over as party leader in 1986, the FPÖ was a party known for its economic liberalism on the one hand and its traditional sympathies with pan-German nationalism on the other (Luther 1987). In that period, the FPÖ generally only obtained 5–8 per cent of the vote. However, following Haider’s ascendance the party never received less than 9.7 per cent of the vote (Figure 1). It achieved its record share of the vote (26.9 per cent) in 1999. Its lowest scores in the past 25 years were in 2002 and 2006, when the party received less than 15 per cent of the vote. These scores followed its participation in a government coalition with the ÖVP as well as Haider’s decision in 2005 to found a new party, the Alliance for the Future of Austria (*Bündnis Zukunft Österreich*, BZÖ), in reaction to internal party disputes. Initially, the BZÖ proved a popular alternative to the FPÖ, yet it has lost much of its support to the FPÖ since Haider’s death just days after the 2008 election. The weakening of the BZÖ was furthered when Haider’s former followers in his stronghold province Carinthia renewed their alliance with the FPÖ in 2010. Recent polls indicate that the FPÖ is again reaching high levels of support under its current leader, Heinz-Christian Strache^4^ Meanwhile, its radical-right rival, the BZÖ, appears now to have become a minor party in electoral terms. As a result we focus solely on the FPÖ in this paper.

The success of the FPÖ has reduced electoral support for the two mainstream parties, the SPÖ and the ÖVP (Müller *et al*. 2004). Since 1986, these two parties have received an average of 37 and 33 per cent of the vote, respectively. In the most recent election in 2008, the incumbent parties SPÖ and ÖVP were only supported by 29.3 and 26.0 per cent of the electorate, respectively. These low scores contrast with the period before the FPÖ’s rise, where both parties regularly received well over 40 per cent of the vote (for more details on changes to the Austrian party system, see Luther 2003; McGann and Kitschelt 2005; Müller *et al*. 2004).

## Radical-Right versus Mainstream Party Supporters: Differences and Similarities

How does the FPÖ fit into, challenge and reshape the previously dominant political divisions in Austria? And how much does its support depend on new political issues, specifically anti-immigration attitudes, Euroscepticism and anti-establishment opinions? In this section we elaborate on our expectations regarding socio-structural divisions as well as old and new issue-based lines of political conflict.

### Established Political Conflicts I: Socio-structural Divisions

Of Lipset and Rokkan’s (1967) famous four socio-structural conflicts, three applied to Austria in the post-war period: the owner–worker, the church–state and the urban–rural cleavages (e.g. Müller 1997; Plasser *et al*. 1992). Importantly, on all three lines of conflict, the ÖVP and the SPÖ took opposing sides (Dolezal 2008). While the SPÖ represented working-class, secular and urban voters, the ÖVP was supported by voters who were close to farming and enterprise interests, were religious (mostly Catholic) and mostly lived in rural areas (Plasser *et al*. 1992). This simple structure of conflict played a substantial role in ensuring the stability of Austria’s post-war party system.

The rise of the FPÖ since 1986 indicates that these pre-existing socio-structural conflict lines no longer have the same ability to structure party competition. This may have resulted from socio-structural changes among the electorate (e.g. an increase in the size by the white-collar middle class) and from the loss of a clear ideological profile by the two mainstream parties (e.g. Evans 2010; Müller *et al*. 2004; Plasser *et al*. 1992). Yet even if socio-structural attributes have lost in importance in determining vote choice, it remains unclear how support for the FPÖ fits into these traditional socio-structural divisions and whether it resembles that of the SPÖ or the ÖVP.

Turning first to the owner–worker divide, there is cross-national evidence that support for radical-right parties is more prevalent among manual workers, low-income service professionals and small-business owners (Evans 2005; Ivarsflaten 2005; McGann and Kitschelt 2005; Oesch 2008; Plasser *et al*. 2000). There has been a significant amount of debate surrounding the argument that voters in these occupational groups are more likely to feel that they are modernization or globalization ‘losers’ (Betz 1994; Kriesi *et al*. 2008; Rydgren 2007) and more likely to experience downward pressure on their wages and a lack of job security (Arzheimer and Carter 2006; Betz 1994; Kessler and Freeman 2005; Lubbers *et al*. 2002). There is indeed some evidence that radical-right voters are more likely to come from the working class (Ivarsflaten 2005; Lubbers *et al*. 2002; Norris 2005; see also Rydgren 2007). Indeed, after Haider became leader of the FPÖ in the mid-1980s, the party followed a conscious strategy of attracting working-class voters (Heinisch 2004). Before then, FPÖ voters were more likely to be self-employed and have higher levels of educational attainment (Härpfer and Gehmacher 1984). We therefore expect FPÖ supporters to resemble SPÖ supporters in that they will be more likely to be from the working-class and low-income groups than ÖVP supporters. Related to social class is the role of education: we expect support for both the SPÖ and the FPÖ to be stronger among groups with less formal education (e.g. Ivarsflaten 2005; Kessler and Freeman 2005; Lubbers *et al*. 2002; Sniderman *et al*. 2000; on Austria see Plasser *et al*. 2007), though the relationship may also be slightly curvilinear, with the strongest support among groups with moderate levels of education (Arzheimer and Carter 2006; Rydgren 2007).^5^

Regarding religion, Arzheimer and Carter (2009) find that the pool of Christian religious voters is generally ‘not available’ for the radical right, since such individuals remain attached to Christian Democratic or Conservative parties. This is supported by the findings of Lubbers *et al*. (2002), who claim that non-religious people (i.e. those with no denomination) are more likely to vote for the radical right. These findings also applied to the FPÖ (Härpfer and Gehmacher 1984), though it should be noted that historically Protestant voters were overrepresented amongst the FPÖ electorate before 1986 (Jagodzinski 1999). We therefore expect FPÖ supporters to be relatively secular and thus again similar to SPÖ supporters. Indeed, existing evidence from Jagodzinski (1999) and Plasser *et al*. (2000) indicates that FPÖ supporters may on average even be *more* secular than SPÖ supporters.

Finally, the SPÖ traditionally has its highest level of support in urban areas, while the ÖVP has always been very strong in Austria’s rural areas due to its links to both the Catholic Church and the farming community. Here, partisan ties remain strong (Plasser *et al*. 2007). Meanwhile, the FPÖ has been characterised as a relatively urban party (Heinisch 2004; Müller *et al*. 2004; Plasser *et al*. 1992). One exception is the mostly rural province Carinthia, which has long been a power base of the FPÖ (and its splinter party, the BZÖ). Overall, we nevertheless expect that FPÖ voters, like those of the SPÖ, will mostly live in urban rather than rural areas.

In sum, we expect that, compared to ÖVP supporters, FPÖ and SPÖ supporters will (1) work in lower-skilled occupational groups and have lower levels of education, (2) be more secular and (3) live mostly in urban areas. Hence, on these three traditional socio-structural divisions related to core historical cleavages, FPÖ supporters should differ from ÖVP supporters but not from SPÖ supporters.

### Established Political Conflicts II: Traditional Ideological Divisions

Based on their sociological foundations, the two mainstream parties in Austria took opposing sides on the country’s two key historical political conflicts: the economy and social and religious values. In contrast, the FPÖ does not position itself very clearly on these topics. On the economy, early studies of the radical right emphasised that these parties had liberal economic views that were to the right of those of many mainstream centre-right parties (Betz 1994; Kitschelt 1995). Indeed, the FPÖ has occasionally defended liberal economic ideas (Heinisch 2003). On the other hand, nowadays radical-right parties tend to take quite centrist positions on economic issues (e.g. Cole 2005; de Lange 2007; Ivarsflaten 2005; Mudde 2007) that resemble those of mainstream centre-right parties (Ivarsflaten 2008). Luther argues that the FPÖ now stresses ‘interventionist economic and social policies targeted at blue-collar voters and welfare state recipients’ (Luther 2009: 1052). Furthermore, on social policy the FPÖ now adopts rather populist positions in favour of the welfare state, though only for native Austrians. Given this lack of clarity and sometimes even fundamental contradiction in FPÖ positions, we do not expect FPÖ supporters to have strong views on economic matters. Indeed, on aggregate they should, if anything, have relatively centrist economic views. In contrast, the supporters of the SPÖ and the ÖVP should still polarise clearly on this dimension (Wagner and Kritzinger 2012).

The supporters of the mainstream parties in Austria have traditionally also been polarised on social and religious values, with traditional values standing in opposition to more liberal ones (de Koster and van der Waal 2007; Kitschelt 1995; Kriesi *et al*. 2008). In this paper, we term this the ‘social liberalism’ dimension. Again, the views of FPÖ supporters on this dimension are not clear. On the one hand, the party is relatively socially conservative. It opposes same-sex civil partnerships as well as strong efforts to increase gender equality (FPÖ 2011: 7). On the other hand, it does not place a lot of emphasis on social values and does not campaign heavily on these matters. In particular, it refers little to religious values. Nevertheless, we would expect FPÖ supporters to have relatively conservative views on the social liberalism dimension, but not views that are particularly extreme within the party system. Thus, the party’s supporters will probably be closer to those of the People’s Party than to those of the SPÖ.

In sum, we therefore expect supporters of the SPÖ and the ÖVP to polarise strongly on the economy and social liberalism. In contrast, FPÖ voters will have an unclear overall position on economic ideology and somewhat conservative (but not particularly extreme) views on social liberalism. On these established ideological lines of conflict, FPÖ support therefore does not fundamentally change the nature of party competition in Austria.

### New Political Conflicts: Opposition to Immigration, the EU and the Political Establishment

Voting for the radical right has increasingly been explained by using individual attitudes such as anti-immigrant views, Euroscepticism and political discontent (Cutts *et al*. 2011; van der Brug 2003; van der Brug and Fennema 2007). These attitudes reflect new political conflicts where the FPÖ opens up new axes of political polarisation. Accordingly, we expect these views to characterise FPÖ supporters and to differentiate them from both SPÖ and ÖVP supporters.

Anti-immigrant sentiment has proven to be an important factor in explaining why people vote for radical-right parties (Cutts *et al*. 2011; Eatwell 1998; Ivarsflaten 2008; Kitschelt 1995; Mughan and Paxton 2006; Norris 2005; van der Brug 2003; for Austria see Bornschier 2012; Dolezal 2008; Plasser *et al*. 2007; Rydgren 2008; though see Lubbers *et al*. 2002). Supporters of these parties want to reduce the number of immigrants coming to their country (Rydgren 2008) and favour a prioritisation of nationals over recent immigrants. Potentially related to this are dissatisfaction with the integration of immigrants as well as anti-Muslim sentiments (though on Islamophobia and radical-right voting, see Cutts *et al*. 2011 and Rydgren 2008). Radical-right parties, including the FPÖ, campaign heavily on this issue and are identified with it (van der Brug and Fennema 2007), while mainstream parties take less extreme positions. Here, it is also important to note the role of the Greens within the Austrian party system. The Austrian Greens campaign heavily on a pro-immigration stance, often taking a position that directly opposes the FPÖ’s views. This may serve to increase the salience of this issue in Austrian politics (for a related argument, see Bale *et al*. 2010).

Eurosceptic views are also a feature of many radical-right parties (Cutts *et al*. 2011; Vasilopoulou 2009), even though they vary in the precise nature and radicalism of this opposition. While mainstream parties are generally in favour of European integration, radical-right parties are often against it because it threatens the nation-state, the defence of which is a central plank of radical-right platforms. Unlike most mainstream parties, they also exploit the perceived nature of the EU as an elite-led, undemocratic project (e.g. Hooghe *et al*. 2002; Ivarsflaten 2008; see below). Here, the FPÖ is typical of other radical-right parties (Dolezal 2008). McGann and Kitschelt (2005) and Plasser *et al*. (2007) have shown that Euroscepticism is associated with support for the radical right in Austria. Together with anti-immigration attitudes, Eurosceptic views may therefore reflect the new importance of a community-based value conflict in political competition (Bornschier 2012) where FPÖ supporters are different from SPÖ and ÖVP supporters.

Finally, scholars consider voting for the radical right to be driven to some extent by dissatisfaction with the mainstream parties and the political system (Cutts *et al*. 2011; Heinisch 2004; Ivarsflaten 2008; Lubbers *et al*. 2002; Plasser *et al*. 2007; Rooduijn *et al.* forthcoming; Rydgren 2005). Radical-right parties present themselves as an alternative that is closer to ‘the people’, cares about ‘true democracy’ and remains uncontaminated by the traditional governing elite (e.g. Rydgren 2005, 2007). In this, radical-right parties may benefit from the increasing prominence of anti-elite sentiments in political discourse (Koopmans *et al*. 2005; Rooduijn *et al.* forthcoming). Indeed, the FPÖ tries hard to exploit this political discontent in its campaigns, as do the Greens. Such appeals may have particular success in Austria, where with the exception of the ÖVP–FPÖ/BZÖ coalition in 2000–2006 the government has been formed by a ‘grand coalition’ between the two mainstream parties SPÖ and ÖVP since 1986. We thus expect FPÖ supporters to be characterised by greater political discontent than supporters of mainstream parties.

### Summary of Expectations

**Figure 2:**
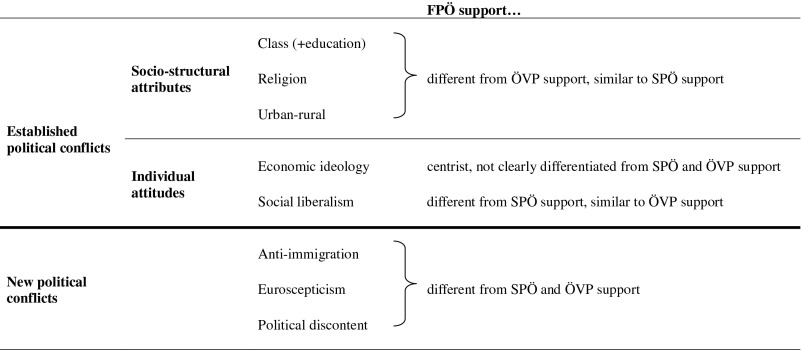
SUMMARY OF EXPECTATIONS

Our expectations for established and new lines of conflict on FPÖ support are summarised in Figure 2.

## Data and Coding

This paper focuses on comparing FPÖ supporters to supporters of its main competitors, the SPÖ and the ÖVP, using the 2009 post-election study carried out by the Austrian National Election Study (AUTNES 2009; Kritzinger *et al*. 2011). The survey was conducted via Computer-Assisted Personal Interviewing (CAPI) in May–June 2009, with a net sample size of 1,165. For more on the 2008 election, see Luther (2009) and Müller (2009).

This survey overcomes some shortcomings that have hampered previous research into why people vote for the FPÖ. For one thing, the AUTNES survey contains a broad set of socio-structural and attitudinal variables that allow us to construct fuller models. The survey also includes propensity-to-vote questions; as explained below, these questions may allow us to construct a more valid measure of party support than direct vote choice or vote intention questions. Furthermore, it offers more information on FPÖ supporters as the FPÖ had returned to a more typical high vote score in the 2008 election.

### Dependent Variable: Party Preference

In this paper, we construct a measure of party support that combines information from questions on the ‘propensity to vote’ (PTV) with that from the question on current vote intention. We use PTV questions, which ask respondents to assess how likely they are ever to vote for a party (van der Eijk *et al*. 2006), to overcome the problem of under-reporting. FPÖ voters are often underrepresented in surveys compared to their actual vote share, possibly due to effects of social desirability. Using the PTV questions as our basic measure provides us with a more realistic proportion of FPÖ supporters in the sample.

We began by coding as party supporters those respondents who only give one party the highest score in the propensity-to-vote question; 75 per cent of respondents fall into this category. We coded cases as ‘missing’ if the party with the highest PTV did not match the one named by the respondent under current vote intention. It is worth noting that, of the respondents who gave the highest PTV to the FPÖ, 26 per cent either gave no valid response (8 per cent) or claimed they had not voted (18 per cent) in the survey question asking about voting behaviour in the 2008 elections. FPÖ supporters may be less reluctant to reveal their party preference in a propensity-to-vote question than in an upfront question about past voting behaviour or current vote intention.

Next, we consider the remaining 25 per cent of respondents who give more than one party the highest PTV rating. When there are such ties for ‘first place’, voter preferences are ambiguous. In these cases, we use the respondent’s current vote intention to code party support. Our final sample contains 312 SPÖ supporters, 323 ÖVP supporters, 153 FPÖ supporters and 87 supporters of the Greens.^6^

### Key Independent Variables

To analyse socio-structural drivers of FPÖ support, we used as independent variables occupational status, education, religious affiliation and residence. We coded occupational status into four categories based on the respondent’s current or prior occupational status: blue-collar; white-collar/civil servants and other public employees; self-employed (with or without employees) or farmers; and ‘other’ (i.e. retirees without formal prior employment status, students and unknown). We do not interpret the ‘other’ category due to its high heterogeneity.^7^

We coded education in three groups, specifically: respondents with compulsory schooling only (or less); respondents with a lower secondary school-leaving certificate or vocational training (equivalent to, e.g., GCSE); and respondents with an upper secondary school-leaving certificate and higher (equivalent to, e.g., A-level and above).

Religiosity was coded using a combination of denominational affiliation and church service attendance. Three groups were coded: respondents who belong to a religious denomination and attend church service at least once a month; respondents who belong to a religious denomination, but attend church less frequently than once a month; and nondenominational respondents.^8^

Residence was measured using the number of inhabitants in the respondent’s administrative region and was coded into three categories: up to 5,000 inhabitants (i.e. villages and small towns); between 5,000 and 50,000 inhabitants (mid-sized towns); and more than 50,000 inhabitants (large towns, cities and Vienna).

In measuring voter attitudes, we followed the recommendations of Heath *et al*. (1994), Ansolabehere *et al*. (2008) and Evans (2010) and constructed multiple-item indicators. The measures of economic views (high scores indicate ‘left-wing’), social liberalism and anti-immigrant views were thus each created based on a series of attitude questions that use 11-point Likert scales. A principal component analysis (PCA) showed that the initial seven attitude items can indeed be separated into three attitude dimensions.^9^ We created the indexes by averaging responses on the three questions.^10^ All three attitude dimensions range between 0 and 10.

The Euroscepticism dimension was made up of questions that measure trust in the EU and support for European integration (Cronbach’s Alpha = 0.85). More specifically, we used four items: trust towards the European Parliament, the Commission and the EU as a whole as well as support of European integration.

To examine attitudinal differences in political discontent at the national level, we created a scale measuring trust in national institutions and views on democracy. The scale was derived from three items: trust in politicians, trust towards the parliament and satisfaction with democracy (Cronbach’s Alpha = 0.70). Both the Euroscepticism and the political discontent scales were rescaled to range between 0 and 1, with higher values indicating less support or lower trust, respectively.

### Control Variables

We added several control variables. We controlled for gender as there is cross-national evidence of a gender gap in radical-right support: men are more likely to vote for the radical right (Betz 1994; Givens 2004; Lubbers *et al*. 2002; Norris 2005). Furthermore, we include age as scholarly work indicates that FPÖ supporters are generally younger than those of other parties (Plasser *et al*. 2007; Wagner and Kritzinger 2012). We also control for whether the respondent has a migration background (i.e. if either the respondent and/or both parents were born abroad) as such citizens should be less likely to support the radical right. Finally, we added a dummy variable for whether the respondent lives in the province of Carinthia; we introduce this control to capture the specific political circumstances of this province, where the FPÖ has long been particularly strong.

## Results

**Figure 3:**
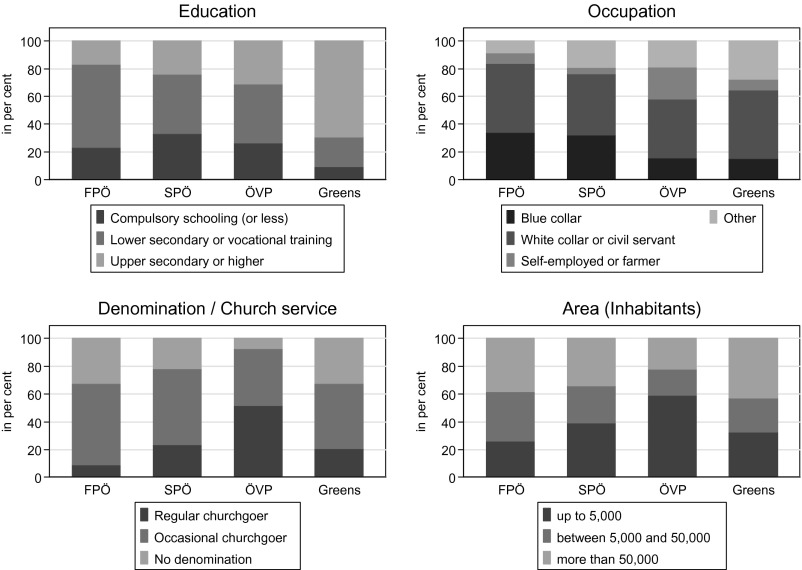
DISTRIBUTION OF SUPPORTERS: SOCIO-STRUCTURAL ATTRIBUTES *Note*: Data from AUTNES (2009). For coding of variables, see text.

We begin our analysis with a brief descriptive analysis of supporters of the FPÖ and the two mainstream parties. In Figure 3, we show the main socio-structural differences between the supporters of these three parties. In terms of education and occupation, we can see that FPÖ supporters are most similar to SPÖ supporters. Only around 20 per cent of their supporters (FPÖ: 17, SPÖ: 24) in our sample have an upper-secondary school-leaving certificate or above, compared to around 31 per cent of ÖVP supporters or 70 per cent of Green supporters. In addition, the two parties have similar proportions of blue-collar supporters, and much higher than among ÖVP supporters. Farmers and self-employed persons are particularly prominent among the ÖVP while a majority of Green supporters are white-collar workers. Regarding religious affiliation, we also find clear-cut patterns: among ÖVP supporters, a very high share attend church services regularly, while among FPÖ and Green supporters a high share of people have no denomination. The SPÖ finds itself in the middle. Finally, turning to the urban–rural divide, we can see quite clearly that FPÖ, SPÖ and Green supporters tend to be quite urban, while ÖVP supporters are primarily found in smaller towns and villages. Chi-square tests show that the differences between the groups of supporters are all statistically significant.

**Figure 4:**
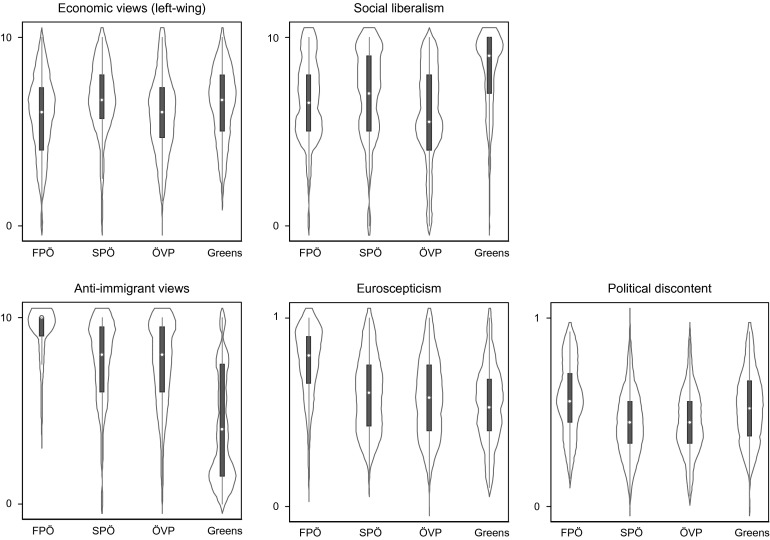
DISTRIBUTION OF SUPPORTERS: ECONOMIC IDEOLOGY, SOCIAL LIBERALISM, AND NEW POLITICAL CONFLICTS *Note*: Data from AUTNES (2009). The violin plots show the median (dot), 25th and 75th percentiles in the box plot format as well as kernel density plots of each variable’s distribution. For coding of variables, see text.

Figure 4 presents violin plots for the established and new attitudinal variables. These show the median and quartiles in the box plot format as well as kernel density plots of each variable’s distribution. The figure shows that FPÖ supporters do not differ much from SPÖ and ÖVP supporters in their economic views and their social liberalism. In general, FPÖ supporters are more similar to ÖVP supporters on economic matters and to SPÖ supporters on social liberalism, though the differences are not great. Finally, Green supporters differ from all other supporters in their social liberalism.

On the new attitudinal lines of conflict, we can see that FPÖ supporters differ very clearly from the supporters of the SPÖ, the ÖVP and the Greens. They are more opposed to immigration and EU integration, and their satisfaction with national political institutions is lower. Concerning immigration, Green supporters also differ substantially from those of the two mainstream parties, as they have far more pro-immigration views.

These bivariate results provide first indications concerning the nature of radical-right support in Austria. To see whether these differences remain statistically significant while controlling for other important predictors, we turn to multivariate analysis. We ran a multinomial logit model (MLM) with four response categories: FPÖ, ÖVP, SPÖ and the Greens. Table 1 presents model results for the socio-structural factors, while Table 2 presents the full model. Each table shows the following comparisons: SPÖ–FPÖ, ÖVP–FPÖ and Green–FPÖ.

**Table:** Table 1. MULTINOMIAL LOGIT MODEL OF PARTY SUPPORT: SOCIO-STRUCTURAL FACTORS

	**SPÖ vs. FPÖ**	**ÖVP vs. FPÖ**	**Greens vs. FPÖ**
*Occupation*			
White collar or civil servant	*Reference category*		
Blue collar	–0.072 (0.288)	–0.823∗ (0.335)	0.461 (0.488)
Self-employed or farmer	–0.864^∗^ (0.384)	0.684^∗^ (0.341)	–0.072 (0.482)
Other	0.357 (0.350)	0.451 (0.366)	0.715^#^ (0.432)
*Education*			
Compulsory schooling (or less)	–0.552 (0.380)	–1.415^∗∗∗^ (0.404)	–3.186^∗∗∗^ (0.589)
Lower secondary or vocational training	–0.503^#^ (0.293)	–0.983^∗∗∗^ (0.298)	–2.441^∗∗∗^ (0.374)
Upper secondary and higher	*Reference category*		
*Denomination / church service*			
Belong / regular service	*Reference category*		
Belong / seldom service	–0.611^#^ (0.332)	–1.698^∗∗∗^ (0.327)	–0.935^∗^ (0.437)
No denomination	–1.041^∗∗^ (0.363)	–2.711^∗∗∗^ (0.387)	–0.611 (0.465)
*Area*			
<= 5,000 inhabitants	0.632^∗^ (0.276)	1.218^∗∗∗^ (0.293)	0.638 (0.374)
<= 50,000 inhabitants	–0.198 (0.259)	–0.158 (0.290)	–0.191 (0.374)
> 50,000 inhabitants	*Reference category*		
*Controls*			
Gender (1 = male)	–0.140 (0.227)	0.024 (0.241)	–0.968^∗∗^ (0.321)
Age	0.035^∗∗∗^ (0.007)	0.036^∗∗∗^ (0.007)	0.005 (0.010)
Migration background (1 = yes)	2.178^∗∗^ (0.762)	2.308^∗∗^ (0.790)	2.331^∗∗^ (0.837)
Carinthia	–1.186^∗^ (0.497)	–0.854^#^ (0.513)	–0.149 (0.645)
*Intercept*	–0.020 (0.528)	0.713 (0.540)	1.443^∗^ (0.683)
Adj. Count R^2^ = 0.29			
*n* = 861			

*Note*: Data from AUTNES (2009). The reference category is supporting the FPÖ. For coding of variables, see text.Standard errors in parentheses. ^#^*p* < 0.10, ^∗^*p* < 0.05, ^∗∗^*p* < 0.01, ^∗∗∗^*p* < 0.001.

**Table:** Table 2. MULTINOMIAL LOGIT MODEL OF PARTY SUPPORT: FULL MODEL

	**SPÖ vs. FPÖ**	**ÖVP vs. FPÖ**	**Greens vs. FPÖ**
*Occupation*			
White collar or civil servant	*Reference category*		
Blue collar	0.172 (0.333)	–0.759^∗^ (0.373)	1.174^∗^ (0.553)
Self-employed or farmer	–0.935^∗^ (0.426)	0.604 (0.382)	–0.637 (0.602)
Other	0.241 (0.387)	0.323 (0.400)	0.599 (0.515)
Education			
Compulsory schooling (or less)	–0.135 (0.430)	–0.860^#^ (0.441)	–2.690^∗∗∗^ (0.688)
Lower secondary or vocational training	0.024 (0.346)	–0.358 (0.343)	–1.598^∗∗∗^ (0.459)
Upper secondary and higher	*Reference category*		
*Denomination / church service*			
Belong / regular service	*Reference category*		
Belong / seldom service	–0.327 (0.359)	–1.253^∗∗∗^ (0.353)	–0.769 (0.491)
No denomination	–0.978^∗^ (0.404)	–2.331^∗∗∗^ (0.426)	–1.134^∗^ (0.550)
*Area*			
<= 5,000 inhabitants	0.915^∗∗^ (0.327)	1.557^∗∗∗^ (0.339)	0.927^∗^ (0.449)
<= 50,000 inhabitants	0.247 (0.306)	0.353 (0.332)	0.366 (0.458)
> 50,000 inhabitants	*Reference category*		
*Attitudes*			
Economic views (left-wing)	0.207^∗∗∗^ (0.059)	0.057 (0.061)	0.165^#^ (0.088)
Social liberalism	–0.001 (0.052)	–0.118^∗^ (0.054)	0.168^∗^ (0.084)
Anti-immigrant views	–0.530^∗∗∗^ (0.090)	–0.437^∗∗∗^ (0.092)	–0.785^∗∗∗^ (0.103)
Euroscepticism	–1.370^#^ (0.824)	–3.110^∗∗∗^ (0.861)	–1.801 (1.137)
Political discontent	–2.910^∗∗∗^ (0.800)	–1.436^#^ (0.833)	–0.108 (1.226)
*Controls*			
Gender (1 = male)	–0.182 (0.260)	–0.052 (0.270)	–1.131^∗∗^ (0.381)
Age	0.037^∗∗∗^ (0.008)	0.036^∗∗∗^ (0.008)	0.024^∗^ (0.012)
Migration background (1 = yes)	1.402^#^ (0.805)	1.487^#^ (0.836)	1.604^#^ (0.912)
Carinthia	–1.291^∗^ (0.548)	–0.694 (0.548)	0.137 (0.695)
*Intercept*	4.749^∗∗∗^ (1.046)	6.731^∗∗∗^ (1.073)	4.913^∗^ (1.412)
Adj. Count R^2^ = 0.39			
*n* = 834			

*Note*: Data from AUTNES (2009). The reference category is supporting the FPÖ. For coding of variables, see text.Standard errors in parentheses. ^#^*p* < 0.10, ^∗^*p* < 0.05, ^∗∗^*p* < 0.01, ^∗∗∗^*p* < 0.001.

Turning first to the socio-structural factors, the results in Table 1 largely confirm the bivariate patterns described above. The similarity of SPÖ and FPÖ supporters in socio-structural attributes is particularly clear for religion and area of residence, and to a lesser extent also for education. Moving from being a regular churchgoer to having no denomination increases the probability of supporting the FPÖ by 25 per cent and that of supporting the SPÖ by 9 per cent, while the probability of supporting the ÖVP declines by 38 per cent.^11^ A person living in a city is 12 per cent more likely to support the FPÖ and 3 per cent more likely to support the SPÖ, but 15 per cent less likely to support the ÖVP. Finally, education is strongly associated with supporting the ÖVP rather than the FPÖ; education is also a strong predictor for Green support compared to the FPÖ. A shift from the highest to the lowest educational group is predicted to lead to a 17 per cent increase in the probability of supporting the FPÖ but also to a 16 per cent increase in the probability of supporting the SPÖ.

For blue-collar workers, the predicted probability of supporting the FPÖ is 28 per cent, for white-collar workers 24 per cent and for the self-employed and farmers 26 per cent. In general, occupation fails to lead to a large shift in predicted probabilities of supporting the FPÖ. The real polarisation here is between SPÖ and ÖVP, where occupation has a very large effect.

In the full model (Table 2) these patterns largely remain consistent, with the significant exception of the effect of education on voting for the ÖVP rather than the FPÖ. Once we control for attitudinal variables, the effect of education is far weaker. This result points to the possibility (also discussed in note 5), that the effect of education on radical-right voting runs through the impact of education on values.

**Figure 5:**
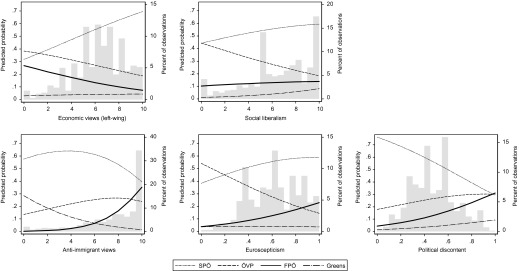
PREDICTED PROBABILITIES BASED ON MULTINOMIAL LOGIT MODEL *Note*: Data from AUTNES (2009). Predicted probabilities calculated using the SPost ado for Stata (see Long and Freese 2006). For coding of variables, see text. See note 11 for values at which control variables were held. Histograms show the distributions of the independent variable.

To understand the precise impact of the added attitudinal variables, we graph differences in predicted probabilities of supporting the four parties for the established and new political conflicts in Figure 5. Turning first to the established ideological conflicts relating to economic and social liberalism (top row), we can see that support for the FPÖ (solid line) and the ÖVP (dashed line) follows an extremely similar pattern. In terms of economic policy positions, support for the ÖVP and the FPÖ are thus similarly structured. In contrast, social liberalism only has a weak effect on supporting the FPÖ, if anything following a similar pattern as support for SPÖ (dotted line). While economic policy views have little impact on the probability of voting for the Greens, social liberalism quite clearly helps to predict Green support (dot-dashed line), also compared to the FPÖ.

Turning to the new attitudinal conflicts (bottom row), the distinctiveness of FPÖ support finally comes to the fore. In all three cases, the drivers of FPÖ support are clearly different from those underlying ÖVP, SPÖ and Green support. Anti-immigration views strongly predict FPÖ support, as the graph and the clear statistical significance of the coefficients show. Interestingly, the Green supporters show the exact opposite trend in the predicted probabilities. The effects for FPÖ support are less strong, but still noteworthy, for both Euroscepticism and national political discontent. The difference of FPÖ supporters to those of the two mainstream parties is always statistically significant, if sometimes at the 0.1 level. This is not as clearly the case for the Green supporters: both on Euroscepticism and political discontent Green supporters are statistically not significantly different from those of the FPÖ.

Regarding our control variables, any differences in migration background fail to achieve common levels of statistical significance. However, we confirm that FPÖ supporters are indeed younger than supporters of the two mainstream parties and more likely to be male than Green supporters. In addition, the FPÖ’s strength in Carinthia mainly seems to damage SPÖ support.

In sum, our analyses show that on established socio-structural conflicts FPÖ supporters resemble SPÖ supporters most. These conflicts distinguish these two parties from ÖVP and Green support even when controlling for extensive attitudinal variables. We can also observe that positions on established political conflicts relating to the economy and social liberalism only weakly structure and characterise FPÖ support. Instead, FPÖ support is strongly predicted by positions on new political conflicts, where extreme values on immigration, the EU and political discontent all strongly explain why citizens support the FPÖ. It is only here that FPÖ supporters have characteristics that differentiate them strongly from those of the two mainstream parties as well as the Greens. On immigration, there is in addition an interesting polarisation between Green and FPÖ support.

## Conclusion

This paper set out to understand how the rise of the FPÖ has weakened and transformed established social and political divisions in Austria. Our aim was to understand how FPÖ support fits into and challenges pre-existing lines of conflict while generating new axes of contestation along formerly ‘less important’ issues. We did so by using a general framework based on established socio-structural and ideological lines of conflict as well as new political divisions.

Our findings show that the support for the FPÖ is directly connected to voters’ positions on new political divisions concerning immigration, European integration and the functioning of the political system. On these issues, FPÖ supporters clearly differ from supporters for other parties. Thus, the rise of the FPÖ provides strong evidence of the declining relevance of traditional cleavage politics (Franklin 2010; Franklin *et al.* 1992) and of the rising importance of new lines of political conflict (Bornschier 2010, 2012; Inglehart and Flanagan 1987; Kriesi 2010; Kriesi *et al*. 2008). Even though the competition for voters between the two mainstream parties is still very strongly structured along familiar social and attitudinal divisions, FPÖ success cannot be explained by these divisions. In other words, a new kind of political polarisation has developed and provides the basis for the electoral success of the FPÖ and the associated electoral decline of the two mainstream parties. In recent research, Wagner and Kritzinger (2012) have also found that positions on socio-cultural issues do not explain how voters choose between mainstream parties. Instead, they help us understand why voters choose new parties, such as the FPÖ and the Greens, rather than established mainstream parties. Interestingly, they could not detect any age group differences, so that new policy considerations can be assumed to be a general driver for FPÖ support (see also van der Brug *et al.* 2012). The FPÖ, and to a certain extent also the Green party, seem to have positioned themselves more clearly on these new and also salient political issues than the two mainstream parties, SPÖ and ÖVP.

Finally, when looking at socio-structural indicators, it appears that the radical right has gained the greatest support among sociological groups previously associated with social democracy. Unlike previous research, which finds Christian Democrats and Conservatives to be in competition with radical-right parties (van der Brug *et al.* 2012), we show that it is indeed Social Democrats that suffer most from radical right success. It seems that these new policy considerations are of particular importance to the portion of the electorate who in the past has formed the socio-structural basis for SPÖ support. It is thus the SPÖ that should be most concerned by polarisation based on these new political conflicts. These findings also shed light on the electoral fortunes of other European Social Democratic parties and how they will be affected by the strength of radical-right parties. In sum, we have found that established social and political divisions have been transformed by, first, the influence of new political conflicts and, second, the weakened socio-structural basis of Social Democratic support.

The finding that FPÖ support depends heavily on these new political divisions may have broader consequences for the re-shaping of political conflict in Europe (see also Kriesi *et al*. 2008). When these new political divisions are strongly salient, electoral competition might be structured mainly around the opposition between mainstream parties on the one hand and parties that mobilise around these new conflicts (such as the FPÖ) on the other. In turn, the extent to which political debates address more established themes will determine whether the polarisation between mainstream parties continues to structure party competition and party support. As a result, our case study of Austria helps us to understand how the Europe of Lipset and Rokkan has changed, but also the extent to which it remains relevant.

## Footnotes

1. We use the term ‘radical right’ to describe the FPÖ throughout this paper. This term has been used frequently in the literature on this party family, including the FPÖ (e.g. Luther 2009; Norris 2005). However, other scholars use terms such as ‘extreme right parties’ (Harrison and Bruter 2011; Lubbers *et al.* 2002), ‘populist right’ (Betz 1994; Betz and Immerfall 1998; Ivarsflaten 2005) or ‘anti-immigration parties’ (Fennema 1997).

2. The List Pim Fortuyn was part of the 2002 coalition in the Netherlands, while Alleanza Nazionale and Lega Nord have been part of governments in Italy. This makes Austria one of only three countries where the radical right has participated in a government coalition (de Lange 2012; Zaslove 2012). A further, unique case is Switzerland, where the Schweizerische Volkspartei (SVP) has also been in government. Radical-right parties have also supported a government without formally joining it in Denmark and Norway.

3. This is not necessarily an exhaustive list of the new issues the FPÖ has made salient to party choice. For example, crime, often linked to radical-right support, has also often dominated the Austrian political agenda. However, as elsewhere this issue has often been linked to immigration in media and public discourse (Dinas and van Spanje 2011) and thus can be subsumed under the issue of ‘immigration’.

4. E.g., At least 19 per cent support for the FPÖ, Gallup telephone survey (*n* = 400), published on 29 June 2013 in the newspaper *Österreich* (available at http://www.österreich.at/nachrichten/Umfrage-SPOe-zieht-davon/108716541 (accessed 10 July 2013).

5. The link between education and the FPÖ vote may also run through values. Higher levels of education would mean that individuals develop more liberal attitudes, specifically in terms of immigration (Arzheimer and Carter 2006; Ivarsflaten 2005; Warwick 1998; Weakliem 2002).

6. BZÖ supporters were excluded due to small sample sizes. We ran a multinomial logit model that also includes BZÖ supporters as possible vote preferences. Results for the key comparisons do not differ substantively.

7. We also considered the more fine-grained categorisation of occupational status suggested by Häusermann (2010). Using this coding scheme did not lead to clearer results concerning the role of occupational status in supporting the radical right.

8. We do not distinguish between religions, so the group of voters with a religious denomination, mainly Catholics, includes Protestants as well as Muslims, though the latter make up less than 1 per cent of our sample.

9. See the Appendix for detailed question wording and PCA results.

10. Cronbach’s Alpha: anti-immigration (Alpha = 0.84), economy (Alpha = 0.55), social liberalism (Alpha = 0.46). The Alpha coefficient for the economy and social liberalism dimensions is rather low. Since this implies a larger amount of measurement error, this will potentially weaken the effects of the attitude dimensions. Nevertheless, we stick to the combination of items for strong theoretical reasons

11. Controls are held at: female, no migration background, lower secondary education, white-collar employee, belong to a religious denomination, but seldom attend church service, residence in community with more than 50,000 inhabitants and not resident in Carinthia. In the full model, attitudinal variables are held at their mean

## Appendix

PRINCIPAL COMPONENT ANALYSIS OF ATTITUDE ITEMS

**Table:**

	**AntImm**	**Econ**	**SocLib**
Immigration to Austria should be significantly curbed.	**0.66**	0.04	–0.01
The state should be tougher toward asylum seekers.	**0.67**	–0.03	0.03
The financial crisis shows that a market economy does not work.	0.14	**0.57**	0.09
A market economy can only function with strong state regulations in place.	0.00	**0.59**	0.06
The current level of social policies should be maintained, even if this means a rise in taxes.	–0.13	**0.56**	–0.18
Same sex marriages should be legally recognised.	–0.23	0.05	**0.62**
Women should be able to decide for themselves whether they want to have an abortion or not.	0.13	–0.02	**0.75**

*Note*: AntImm ‘Anti-immigrant views’; Econ ‘Economic views’; SocLib ‘Social liberalism’; Varimax rotation; listwise deletion; *n* = 704; Loadings >|0.50| are set in bold; KMO = 0.57.
